# Postoperative acute kidney injury requiring continuous renal replacement therapy and outcomes after coronary artery bypass grafting: a nationwide cohort study

**DOI:** 10.1186/s13019-021-01704-7

**Published:** 2021-10-26

**Authors:** Tak Kyu Oh, In-Ae Song

**Affiliations:** Department of Anesthesiology and Pain Medicine, Seoul National University Bundang Hospital, 166 Gumi-ro, Bundang-gu, Seongnam, 463-707 Korea

**Keywords:** Acute kidney injury, Cardiovascular, CRRT, Coronary artery bypass grafting

## Abstract

**Background:**

Previous studies reported that patients with acute kidney injury (AKI) requiring continuous renal replacement therapy (CRRT) after cardiac surgery were at a higher risk of postoperative mortality. However, the impact of AKI and CRRT on long-term mortality has not yet been identified. Therefore, we investigated whether postoperative AKI requiring CRRT was associated with one-year all-cause mortality after coronary artery bypass grafting (CABG).

**Methods:**

For this population-based cohort study, we analyzed data from the National Health Insurance Service database in South Korea. The cohort included all adult patients diagnosed with ischemic heart disease who underwent isolated CABG between January 2012 and December 2017.

**Results:**

A total of 15,115 patients were included in the analysis, and 214 patients (1.4%) required CRRT for AKI after CABG during hospitalization. They received CRRT at 3.1 ± 8.5 days after CABG, for 3.1 ± 7.8 days. On multivariable Cox regression, the risk of 1-year all-cause mortality in patients who underwent CRRT was 7.69-fold higher. Additionally, on multivariable Cox regression, the 30-day and 90-day mortality after CABG in patients who underwent CRRT were 18.20-fold and 20.21-fold higher than the normal value, respectively. Newly diagnosed chronic kidney disease (CKD) requiring renal replacement therapy (RRT) 1 year after CABG in patients who underwent CRRT was 2.50-fold higher. In the generalized log-linear Poisson model, the length of hospital stay (LOS) in patients who underwent CRRT was 5% longer.

**Conclusions:**

This population-based cohort study showed that postoperative AKI requiring CRRT was associated with a higher 1-year all-cause mortality after CABG. Furthermore, it was associated with a higher rate of 30-day and 90-day mortality, longer LOS, and higher rate of CKD requiring RRT 1 year after CABG. Our results suggest that CRRT-associated AKI after CABG may be associated with an increased risk of mortality; hence, there should be interventions in these patients after hospital discharge.

**Supplementary Information:**

The online version contains supplementary material available at 10.1186/s13019-021-01704-7.

## Background

Acute kidney injury (AKI), defined as the rapid deterioration of kidney function, occurs in 2%–18% of all hospitalized patients [1–3]. Approximately 40% of total AKI cases among inpatients occur after surgical procedures [4]. AKI can delay recovery and increase hospital mortality and costs [5, 6]. Postoperative AKI is more common after cardiac surgery than other types of surgery [7], with an incidence of up to 30% [8]. Since the occurrence of AKI after cardiac surgery is associated with poorer long-term survival [9], the management of postoperative AKI has been an important issue and is considered a major clinical challenge in the management of patients undergoing cardiac surgery [10, 11].

Continuous renal replacement therapy (CRRT) has been used to treat patients with severe AKI [12, 13]. The incidence of AKI requiring CRRT after cardiac surgery is reported to be 1.0%–2.0% [14, 15], and patients with severe AKI requiring CRRT are reported to have higher postoperative mortality [15, 16]. A previous study reported in-hospital mortality as high as 43.5% in patients who required CRRT after cardiac surgery [17]. However, the relationship between relative long-term survival and postoperative AKI requiring CRRT has not been definitively identified, and further studies are needed. Additionally, the association between postoperative AKI requiring CRRT and various outcomes such as 30-day mortality, 90-day mortality, length of hospital stay (LOS), and newly diagnosed chronic kidney disease (CKD) requiring renal replacement therapy (RRT) after cardiac surgery has not been investigated in detail.

Therefore, this study aimed to investigate whether postoperative AKI requiring CRRT was associated with 1-year all-cause mortality after coronary artery bypass grafting (CABG), which is one of the most common cardiac surgical procedures [18]. Additionally, we investigated whether postoperative AKI requiring CRRT was associated with various negative outcomes after CABG.

## Methods

### Design and ethical statements

This population-based cohort study was conducted according to the Strengthening of Reporting of Observational Studies in Epidemiology guidelines and Population-Exposure-Comparator-Outcome (PECO) guidelines [19, 20]. The study protocol was approved by the Institutional Review Board of Seoul National University Bundang Hospital (X-1908-556-901) and the Health Insurance Review and Assessment Service (NHIS-2019-1-505). This study was conducted in accordance with the guidelines of the Declaration of Helsinki. The requirement for informed consent was waived because the data analyses were performed retrospectively using anonymous data derived from the South Korean National Health Insurance Service (NHIS) database.

### Data source

We used health records obtained from the NHIS database. In South Korea, all disease diagnoses and prescription information regarding drugs and/or procedures provided under the public insurance system should be registered with the NHIS database. The study data were extracted by an independent medical record technician at the NHIS center who had no conflicts of interest relevant to this study. Data extraction was performed on November 1, 2019.

### Study population

We included all adult patients diagnosed with ischemic heart disease (International Classification of Diseases [ICD]-10 codes I20*–I25*) who underwent isolated CABG between January 2012 and December 2017. We excluded patients who 1) underwent repeat CABG surgeries, 2) were under the age of 18 years (i.e., pediatric patients), 3) did not have complete medical records, and 4) had any history of RRT before CABG (i.e., hemodialysis or peritoneal dialysis).

### Exposure: CRRT after CABG

Patients who received CRRT for AKI during hospitalization after CABG were classified into the CRRT group. The remaining patients who did not undergo CRRT during hospitalization after CABG were classified into the control group.

### Study endpoints (outcomes)

The primary endpoint of this study was 1-year all-cause mortality after CABG, which was defined as all-cause mortality occurring within 1 year of CABG. The secondary endpoints were 30-day and 90-day mortality after CABG, LOS after CABG, and newly diagnosed CKD requiring RRT after CABG, which was evaluated 1 year after the date of CABG.

### Confounders

We used the following variables as confounders: 1) demographic variables (age and sex), 2) socioeconomic variables (residence at the time of CABG [capital city/other metropolitan city/other area] and income level at the time of CABG [four groups in quartile ratio]), 3) the Charlson comorbidity index at the time of CABG, which was calculated using registered ICD-10 diagnostic codes (Additional file 1) from 1 year before CABG to the date of CABG; 4) use of an intraoperative cardiopulmonary bypass pump (off-pump CABG /on-pump CABG); 5) the number of coronary artery grafts (1 or ≥ 2); 6) absolute hospital volume as the total number of hospital beds (four groups in quartile ratio: Q1 ≤ 904, 905 ≤ Q2 ≤ 1119, 1120 ≤ Q3 ≤ 2413, and 2414 ≤ Q4); 7) emergency or urgent CABG; and 7) the year in which CABG was performed.

### Statistical analyses

The demographic, socioeconomic, and clinical information between the CRRT and control groups were compared using the *t*-test for continuous variables and the chi-square test for categorical variables. First, we performed univariate and multivariate Cox regression analyses to investigate the hazard of 1-year all-cause mortality after CABG. All covariates were included in the multivariate model for the adjustment. We then performed three multivariable Cox regression analyses with two secondary endpoints using the landmark method (30-day mortality and 90-day mortality). The duration from the date of CABG to the date of death or 30-day, 90-day, and 1-year were used as time, while death within 30-day, 90-day, and 1-year after CABG were set as events in the time-to-event analyses. The results of Cox regression analysis were presented as hazard ratios (HRs) with 95% confidence intervals (CIs). Log–log plots were used to confirm that the central assumption of the Cox proportional hazard models was satisfied, and there was no multicollinearity with a variance inflation factor of < 2.0. C-statistics were used to investigate the predictive ability of the multivariable Cox regression model with respect to 1-year all-cause mortality, and the results were presented as C-index with 95% CI [21].

We subsequently conducted a multivariable logistic regression analysis for newly diagnosed CKD requiring RRT one year after CABG. All covariates were included in the multivariable logistic regression models for adjustment, and the results are presented as odds ratios (ORs) with 95% CIs. We fitted a GLM with all covariates as another secondary endpoint of LOS. For the GLM, a Poisson distribution was assumed for the dependent variable (LOS), and the log-link function was used. The results of GLM are presented as exponentiated regression coefficients with 95% CIs. All statistical analyses were performed using SAS (version 9.4; SAS Institute Inc., Cary, NC, USA) and R software (version 3.6.2; R Foundation for Statistical Computing, Vienna, Austria). *Statistical significance was set at P* < 0.05.

## Results

**Fig. 1:**
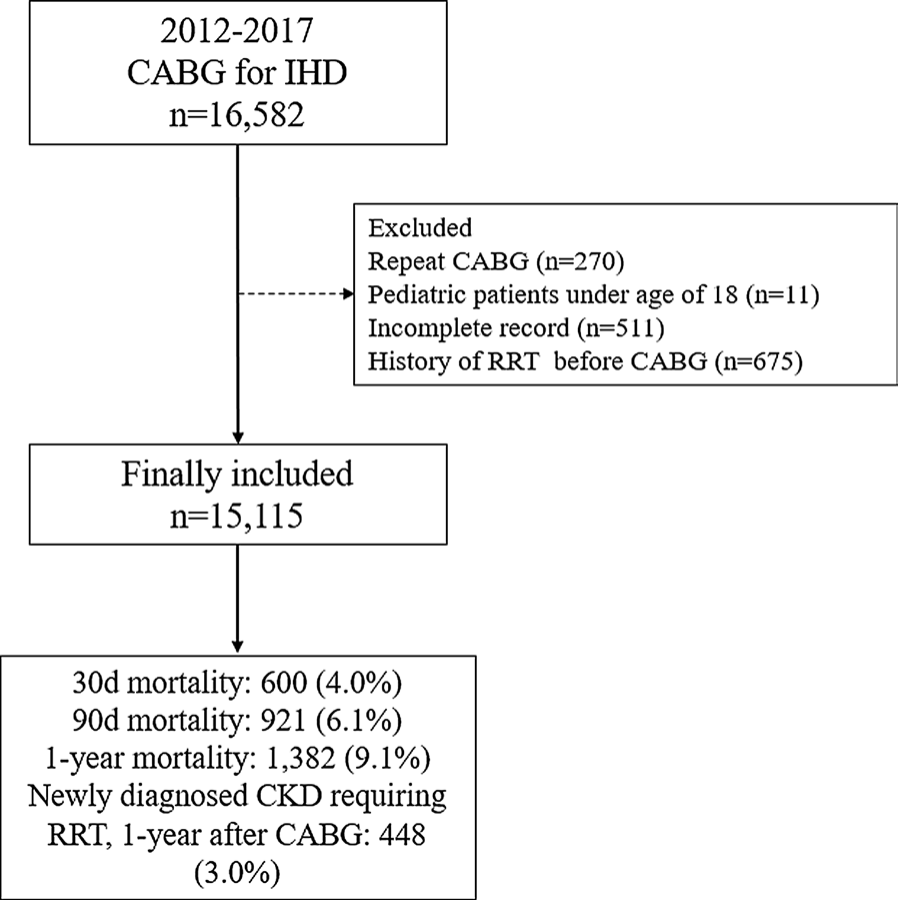
Flowchart of the study design. *CABG* coronary artery bypass grafting; *IHD* ischemic heart disease; *RRT* renal replacement therapy

A total of 16,582 patients who underwent isolated CABG for the treatment of ischemic heart disease between January 2012 and December 2017 were initially screened. Among them, we excluded 270 patients who underwent repeat CABG surgery, 11 were younger than 18 years, 511 had incomplete medical records, and 675 patients who had any history of RRT before CABG. Finally, 15,115 patients were included in the analysis (Fig. 1). Among them, 214 patients (1.4%) underwent CRRT for AKI after CABG during hospitalization. They received CRRT at 3.1 ± 8.5 days after CABG for 3.1 ± 7.8 days during hospitalization. Among the 15,115 patients, 30-day, 90-day, and 1-year all-cause mortality after CABG occurred in 600 (4.0%), 921 (6.1%), and 1,382 (9.1%) patients, respectively. Additionally, 448 (3.0%) patients were diagnosed with CKD requiring RRT 1 year after CABG. Table 1 shows the results of the comparison of demographic, socioeconomic, and clinical information between the CRRT and control groups.

**Table 1:** Comparision of the demographic, socioeconomic, and clinical information between CRRT group and control group

Variable	CRRT (n = 214)	Control (n = 14,901)	*P*-value
Age, year	69.4 (10.6)	65.6 (10.2)	< 0.001
Sex, Male	124 (57.9)	10,951 (73.5)	< 0.001
Residence at CABG			0.768
Capital city	45 (21.0)	3399 (22.8)	
Other metropolitan city	38 (17.8)	2730 (18.3)	
Other area	131 (61.2)	8772 (58.9)	
Income level at CABG			0.073
Q1	42 (19.6)	2679 (18.0)	
Q2	52 (24.3)	3528 (23.7)	
Q3	32 (15.0)	3320 (22.3)	
Q4	88 (41.1)	5374 (36.1)	
Charlson comorbidity index at CABG			< 0.001
0–3	23 (10.7)	3264 (21.9)	
4–5	59 (27.6)	4060 (27.2)	
≥ 6	132 (61.7)	7577 (50.8)	
Intraoperative CPB use			< 0.001
Off Pump CABG	43 (20.1)	8011 (53.8)	
On Pump CABG	171 (79.9)	6890 (46.2)	
The number of coronary artery graft			< 0.001
1	51 (23.8)	2405 (16.1)	
≥ 2	163 (76.2)	12,496 (83.9)	
Total number of hospital bed			< 0.001
Q1 ≤ 904	65 (30.4)	3723 (25.0)	
905 ≤ Q2 ≤ 1119	70 (32.7)	3870 (26.0)	
1120 ≤ Q3 ≤ 2413	62 (29.0)	4547 (30.5)	
2414 ≤ Q4	17 (7.9)	2761 (18.5)	
Length of hospital stay for CABG, day	18.0 (10.6)	15.5 (7.9)	< 0.001
Newly diagnosis of CKD requiring RRT	17 (7.9)	431 (2.9)	< 0.001
Urgent or Emergency CABG	34 (15.9)	2464 (16.5)	0.800
The year of CABG			0.883
2012	31 (14.5)	2450 (16.4)	
2013	29 (13.6)	2183 (14.7)	
2014	37 (17.3)	2384 (16.0)	
2015	41 (19.2)	2889 (19.4)	
2016	45 (21.0)	3168 (21.3)	
2017	31 (14.5)	1827 (12.3)	

*CABG* coronary artery bypass grafting; *CRRT* continuous renal replacement therapy; *CPB* cardiopulmonary bypass; *CKD* chronic kidney disease; *RRT* renal replacement therapy

### One-year all-cause mortality after CABG

**Fig. 2:**
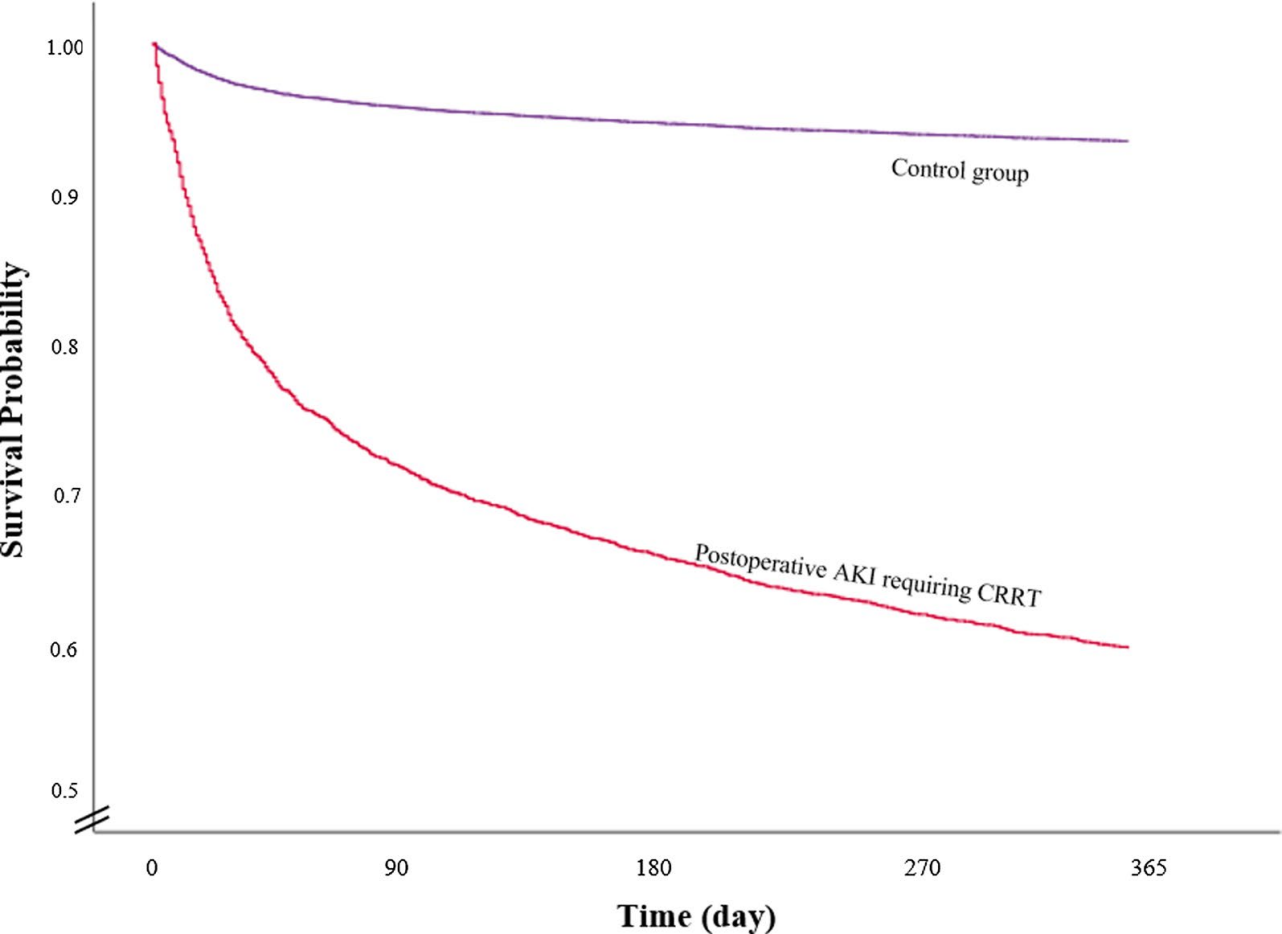
Analysis of survival after isolated CABG. The plot of survival probability after CABG was derived from a multivariable Cox regression model. *CABG* coronary artery bypass grafting; *CRRT* continuous renal replacement therapy

Additional file 2 and Table 2 show the results of univariate and multivariate Cox regression analyses for 1-year all-cause mortality after CABG. In the multivariable model, the risk of 1-year all-cause mortality in patients who were exposed to CRRT was 7.69-fold higher (HR, 7.69; 95% CI: 6.54 to 9.05; *P* < 0.001) than that in the control group. The survival plot derived from the multivariable Cox regression model showed a similar tendency, as shown in Fig. 2. The C-index of the multivariable model was 0.83 (95% CI: 0.81 to 0.84).

**Table 2:** Mutlviariable Cox regression analysis for 1-year all-cause mortality after CABG

Variable	Multivariable model	*P*-value
	HR (95% CI)	
CRRT	7.69 (6.54, 9.05)	< 0.001
Age, year	1.06 (1.05, 1.07)	< 0.001
Sex, Male	1.07 (0.95, 1.20)	0.258
Residence at CABG		
Capital city Capital city	1	
Other metropolitan city	1.00 (0.85, 1.19)	0.977
Other area	1.05 (0.91, 1.20)	0.525
Economic status at CABG		
Q1	1	
Q2	1.07 (0.90, 1.26)	0.460
Q3	0.99 (0.83, 1.17)	0.880
Q4	0.93 (0.80, 1.09)	0.356
Total hospital bed number		
Q1 ≤ 904	1	
905 ≤ Q2 ≤ 1119	1.07 (0.93, 1.22)	0.369
1120 ≤ Q3 ≤ 2413	0.99 (0.83, 1.17)	0.880
2414 ≤ Q4	0.93 (0.80, 1.09)	0.356
Charlson comorbidity index at CABG		
0–3	1	
4–5	0.98 (0.82, 1.17)	0.832
≥ 6	1.22 (1.04, 1.43)	0.014
Urgent or Emergency CABG	0.96 (0.83, 1.10)	0.521
Intraoperative CPB use		
Off Pump CABG	1	
On Pump CABG	1.95 (1.73, 2.19)	< 0.001
The number of coronary artery graft The number of coronary artery graft		
1	1	
≥ 2	0.69 (0.61, 0.78)	< 0.001
The year of CABG		
2012	1	
2013	0.98 (0.81, 1.17)	0.786
2014	0.86 (0.71, 1.03)	0.104
2015	0.83 (0.70, 1.00)	0.044
2016	0.83 (0.70, 0.99)	0.038
2017	0.78 (0.64, 0.95)	0.015

C-index 0.83 (0.81, 0.84)

*CABG* coronary artery bypass grafting; *HR* hazard ratio; *CI* confidence interval; *CRRT* continuous renal replacement therapy; *CPB* cardiopulmonary bypass; *CKD* chronic kidney disease; *RRT* renal replacement therapy

### Analysis for secondary endpoints

Table 3 shows the results of the multivariable models for the secondary endpoints. The 30-day and 90-day mortality rates after CABG in patients who underwent CRRT were 18.20-fold (HR: 18.20, 95% CI: 13.01–24.71; *P* < 0.001) and 20.21-fold (HR: 20.21, 95% CI: 14.35–29.20; *P* < 0.001) higher than that of the control group, respectively. Newly diagnosed CKD requiring RRT, 1 year after CABG in patients who underwent CRRT was 2.50-old (OR: 2.50, 95% CI: 1.49 to 4.19; *P* = 0.001) higher than that of the control group. The generalized linear regression model (GLM) for LOS showed that the LOS in patients who were exposed to CRRT was 5% longer (exponentiated regression coefficient: 1.05, 95% CI: 1.02 to 1.08; *P* = 0.004) than that of the control group.

**Table 3:** Multivariable models for secondary endpoints

Variable	Multivariable model	*P*-value
	HR, OR or Exp.Coef (95% CI)	
30-day mortality after CABG (HR)		
CRRT	18.20 (13.01, 24.71)	< 0.001
90-day mortality after CABG (HR)		
CRRT	20.21 (14.35, 29.20)	< 0.001
Newly diagnosed CKD requiring RRT, 1 year after CABG (OR)		
CRRT	2.50 (1.49, 4.19)	0.001
LOS after CABG (Exp.Coef)		
CRRT	1.05 (1.02, 1.08)	0.004

*HR* hazard ratio; *OR* odds ratio; *Exp. Coef,* exponentiated regression coefficient; *CI* confidence interval; *CABG* coronary artery bypass grafting; *CRRT* continuous renal replacement therapy; *CKD* chronic kidney disease; *RRT* renal replacement therapy; *LOS* length of hospital stay

## Discussion

This population-based cohort study in South Korea showed that patients who developed AKI requiring CRRT after CABG had higher 1-year all-cause mortality. Furthermore, these patients also had longer LOS, higher 30-day and 90-day mortality, and a higher incidence of newly diagnosed CKD requiring RRT 1 year after CABG. Our results suggest that CRRT-associated AKI after CABG is associated with an increased risk of long-term mortality and persistent kidney injury requiring RRT. Although previous studies reported that patients with AKI requiring CRRT after cardiac surgery were at higher risk of postoperative mortality [15–17], its association with relative long-term outcomes such as 1-year mortality or persistent kidney injury requiring RRT at 1-year after CABG has not yet been reported. Therefore, our novel results suggest that severe postoperative AKI requiring CRRT might affect long-term outcomes (up to 1-year) after CABG.

For this study, we excluded patients with any history of RRT before CABG to focus on the effect of newly developed AKI requiring CRRT after CABG. In a retrospective cohort study by Pistolesi et al., severe postoperative AKI requiring CRRT was associated with higher 30-day and in-hospital mortality rates after cardiac surgery [22]. Another retrospective case–control study by Perez-Valdivieso et al. reported that severe postoperative AKI after cardiac surgery was associated with higher in-hospital mortality after CABG [23]. Other older studies have also stated that postoperative AKI requiring CRRT was an independent prognostic factor for higher mortality in patients undergoing cardiac surgery [15–17]. Our study revealed that a relatively long-term outcome (i.e., 1-year all-cause mortality) was negatively associated with AKI requiring CRRT after CABG. While the previous cohort studies analyzed relatively small sample sizes in a single institution [15–17, 22, 23], the sample size used in our study was very large, as we searched the entire national health database of South Korea.

Our finding regarding newly diagnosed CKD requiring RRT 1 year after CABG was the novel outcome of this study. Since the development of CKD is a well-known complication and sequelae among patients with AKI [24, 25], newly diagnosed CKD in these patients is an important clinical issue. A systemic review and meta-analysis of nine observational studies by Corredor et al. reported a higher long-term mortality risk in patients with persistent renal dysfunction compared to those in whom renal function recovers to baseline levels before discharge from the hospital [26]. Similarly, another cohort study by Swaminathan et al. reported that early recovery of renal function in patients who developed AKI after cardiac surgery was associated with improved long-term survival [27]. However, all these studies focused on the effect of postoperative AKI on long-term mortality after cardiac surgery, not on newly developed CKD requiring RRT after AKI. Considering that the cohort in our study had no history of RRT before CABG, our results suggest that AKI requiring CRRT after CABG was associated with a higher risk of developing end-stage renal disease after discharge from the hospital.

Our study has some limitations that should be addressed. First, we were unable to include some important physiological variables, such as body mass index at the time of CABG because they were not included in the NHIS database. Second, some important operative characteristics, such as the preoperative American Society of Anesthesiologists’ physical status, duration of surgery, and anesthesia used for CABG, were not evaluated. Again, this was because these data were not included in the NHIS database. Third, we defined comorbidities using the ICD-10 codes registered in the NHIS database to calculate the Charlson comorbidity index, as shown in Additional file 1. However, these diseases registered according to the ICD-10 codes might have differed from the actual comorbidities present in the patients. Fourth, our adjustment for the multivariable analysis was controlled using only known confounders. Residual or unmeasured confounders may have affected the results of the analysis. Fifth, although we used the Charlson comorbidity index to reflect the comorbidity status at CABG, we were unable to evaluate other important comorbidity status parameters in detail. For example, we could not evaluate cardiac function in detail because this information was not included in the NHIS database. Lastly, patients requiring CRRT after CABG had AKI in this study. However, there might be non-renal causes of CRRT use after CABG. These include rhabdomyolysis or sepsis. Although sepsis or rhabdomyolysis was not a direct cause of CRRT, they could also result in the development of AKI, such as sepsis- [28] or rhabdomyolysis-associated AKI [29]. Therefore, AKI requiring CRRT after CABG may occur due to AKI, which is directly or indirectly associated with other pathologic conditions.

## Conclusion

This population-based cohort study in South Korea showed that the development of postoperative AKI requiring CRRT was associated with higher 1-year all-cause mortality after CABG. Furthermore, it was associated with higher 30-day and 90-day mortality, longer LOS, and a higher rate of newly diagnosed CKD requiring RRT 1 year after CABG. Our results suggest that patients who undergo CRRT-associated AKI after CABG may be a high-risk group that needs intervention after hospital discharge.

## Supplementary Information

**Additional file 1**. ICD-10 codes

**Additional file 2**. Univariable Cox regression analysis for 1-year all-cause mortality after CABG.

## Data Availability

The data that support the findings of this study are available from the National Health Insurance System, but restrictions apply to the availability of these data, which were used under license for the current study and so are not publicly available. However, data are available from the authors upon reasonable request and with permission from the National Health Insurance System (https://nhiss.nhis.or.kr/bd/ab/bdaba000eng.do).

## Abbreviations

AKI: Acute kidney injury
CRRT: Continuous renal replacement therapy
CABG: Coronary artery bypass grafting
CKD: Chronic kidney disease
LOS: Length of hospital stay
RRT: Renal replacement therapy
NHIS: National Health Insurance Service
ICD: International Classification of Diseases
