# Supplementary material for: Postoperative acute kidney injury requiring continuous renal replacement therapy and outcomes after coronary artery bypass grafting: a nationwide cohort study
Source: J Cardiothorac Surg. 2021 Oct 26;16:315. doi: 10.1186/s13019-021-01704-7 (PMC8549378; doi:10.1186/s13019-021-01704-7)
Supplement: Supplementary file 2 — Additional file 2. Univariable Cox regression analysis for 1-year all-cause mortality after CABG. [file 13019_2021_1704_MOESM2_ESM.docx]

Additional file 2. Univariable Cox regression analysis for 1-year all-cause mortality after CABG

| Variable | | Univariable model | P-value |
| --- | --- | --- | --- |
|  |  | HR (95% CI) |  |
| CRRT | | 10.84 (9.24, 12.72) | <0.001 |
| Age, yr | | 1.06 (1.06, 1.07) | <0.001 |
| Sex, Male | | 0.72 (0.64, 0.80) | <0.001 |
| Residence at CABG | |  |  |
|  | Capital city | 1 |  |
|  | Other metropolitan city | 1.13 (0.96, 1.34 | 0.142 |
|  | Other area | 1.13 (0.99, 1.29) | 0.077 |
| Economic status at CABG | |  |  |
|  | Q1 | 1 |  |
|  | Q2 | 1.08 (0.92, 1.28) | 0.359 |
|  | Q3 | 0.99 (0.83, 1.17) | 0.896 |
|  | Q4 | 1.07 (0.92, 1.25) | 0.366 |
| Total hospital bed number | |  |  |
|  | Q1≤904 | 1 |  |
|  | 905≤Q2≤1119 | 1.12 (0.98, 1.28) | 0.102 |
|  | 1120≤Q3≤2413 | 0.85 (0.74, 0.97) | 0.018 |
|  | 2414≤Q4 | 0.49 (0.41, 0.60) | <0.001 |
| Charlson comorbidity index at CABG | |  |  |
|  | 0-3 | 1 |  |
|  | 4-5 | 1.28 (1.07, 1.53) | 0.007 |
|  | $\geq$6 | 1.88 (1.61, 2.19) | <0.001 |
| Intraoperative CPB use | |  |  |
|  | Off Pump CABG | 1 |  |
|  | On Pump CABG | 2.40 (2.14, 2.68) | <0.001 |
| Urgent or Emergency CABG | | 0.96 (0.83, 1.10) | 0.519 |
| The number of coronary artery graft | |  |  |
|  | 1 | 1 |  |
|  | $\geq$2 | 0.59 (0.52, 0.67) | <0.001 |
| The year of CABG | |  |  |
|  | 2012 | 1 |  |
|  | 2013 | 0.99 (0.82, 1.19) | 0.907 |
|  | 2014 | 0.89 (0.74, 1.07) | 0.202 |
|  | 2015 | 0.90 (0.76, 1.07) | 0.235 |
|  | 2016 | 0.88 (0.74, 1.05) | 0.153 |
|  | 2017 | 0.91 (0.75, 1.11) | 0.358 |

CABG, coronary artery bypass grafting; HR, hazard ratio; CI, confidence interval; CRRT, continuous renal replacement therapy; CPB, cardiopulmonary bypass; CKD, chronic kidney disease; RRT, renal replacement therapy
